# Navigating dietary advice for multiple sclerosis

**DOI:** 10.1111/hex.13226

**Published:** 2021-04-10

**Authors:** Rebecca D. Russell, Lucinda J. Black, Andrea Begley

**Affiliations:** ^1^ School of Population Health Curtin University Perth WA Australia

**Keywords:** autonomy, diet, dietary modifications, focus groups, motivation, multiple sclerosis, qualitative, self‐determination theory

## Abstract

**Background:**

Multiple sclerosis (MS) is an inflammatory demyelinating disease with no known cure. Numerous diets are promoted to reduce symptoms or even cure MS, despite insufficient evidence for any therapeutic diet. There are few qualitative studies exploring the experiences of people with MS in relation to diet, and no use of theory to explain the findings.

**Purpose:**

To explore the experiences of adults with MS when navigating dietary advice, their attitudes when making dietary decisions, and their needs regarding dietary resources and education.

**Methods:**

In this qualitative study, we conducted six focus groups with people with MS (n = 33 plus one spouse without MS). Groups were audio‐recorded and transcribed verbatim. Primary analysis used a general inductive approach with thematic analysis. Secondary analysis aligned themes with the constructs of the self‐determination theory.

**Results:**

Six themes emerged: (a) confusion about where to seek dietary advice; (b) scepticism towards national dietary guidelines; (c) personalized approaches to dietary change; (d) barriers to dietary changes; (e) judging if dietary changes work; and (f) wanting dietary guidelines for MS.

**Conclusion:**

People with MS are highly motivated to make dietary changes and improve their health. The self‐determination theory explained why people with MS make dietary modifications, and the varying levels of motivation. MS‐specific dietary resources and nutrition education need to incorporate ways to increase autonomous forms of motivation. Future dietary intervention studies could use the self‐determination theory as a framework to improve long‐term adherence to healthier diets.

## 1 INTRODUCTION

Multiple sclerosis (MS) is an inflammatory demyelinating disease of the central nervous system with no known cure.^1^ Immune‐mediated attacks cause inflammation and damage to the myelin sheaths, interrupting nerve signal transmission.^2^ Any of the sensory, visual, or motor systems can be affected, causing symptoms that vary widely between individuals and over time.^3^ MS affects more than 25 000 Australians, and approximately 2.3 million people worldwide; three‐quarters of those are female.^3^ The most common form of MS is relapsing‐remitting MS, where periods of neurological decline are followed by periods of remission.^4^ Over time this may progress to secondary progressive MS (half of cases over 10 years^5^), where deterioration is ongoing. Less common is primary progressive MS (occurring in 10%‐15% of cases), where deterioration is from the onset, and there are no remissions.^4^

Although diet has been proposed as a potential modifiable risk factor to reduce MS symptom severity and/or slow disability progression,^6^ there is insufficient evidence to support any specific therapeutic diets.^7^ As such, the dietary advice for people with MS (pwMS) is to follow Government‐issued national dietary guidelines. This is to reduce the risk of comorbid diseases (such as cardiovascular disease and type 2 diabetes) and ensure optimum nutritional status. This is imperative, as vascular co‐morbidities have been associated with increased disability progression,^8^ and some nutrient deficiencies can exacerbate symptoms and accelerate demyelination.^9^ The food group and nutrient intake recommendations in the Australian Dietary Guidelines (an example of national dietary guidelines) can be achieved with a range of dietary patterns, including vegetarian, vegan and Mediterranean diets.^10^ Unfortunately, less than 4% of Australians achieve these food group and nutrient recommendations.^11^ To our knowledge, there is no literature reporting how many pwMS follow national dietary guidelines.

There are numerous non–evidence‐based diets promoted online, claiming to reduce MS symptoms, slow MS progression, or cure MS.^12^ This creates a challenge for pwMS when deciding what foods to eat, given that the diets are often contradictory^12^ and restrictive, that is they don't meet the minimum nutrient requirements outlined in national dietary guidelines. There is an opportunity to provide tailored education to assist pwMS in decision making and meal planning in order to improve dietary intakes. Dietary education programs need to take into account factors such as food preferences, budgets, and food literacy skills.^13^

Quantitative studies show that more than 80% of pwMS consider diet to be important,^14^ and around 40% report making dietary modifications after their diagnosis.^14^, ^15^, ^16^, ^17^ Reducing symptoms or number of relapses, losing weight, having a sense of control, slowing disease progression and curing themselves of MS are common reasons why pwMS make dietary modifications.^14^, ^18^ The most common dietary changes described are adopting a low‐fat^15^, ^16^, ^17^ or low‐carbohydrate diet,^17^ modifying fatty acid intake,^14^ eliminating meat intake,^14^ decreasing sugar intake,^16^ and increasing fruit and/or vegetable consumption.^15^ Such modifications are not always evidence‐based, or in line with national dietary guidelines. While there is literature capturing what specific dietary changes are made by pwMS, little is known about why pwMS make non–evidence‐based dietary modifications, or what would motivate them to increase adherence to a healthy diet.

Only two qualitative studies have explored the rationale behind the dietary modifications made by pwMS. Fatigue and limited mobility have been reported as barriers to engaging in healthy dietary behaviours.^19^ In our previous study, people recently diagnosed with MS expressed that a lack of dietary advice from neurologists was incompatible with the seriousness of the disease, and experimented with dietary modifications to control or cure their MS.^18^ There has been little theoretical explanation as to why pwMS make and adhere to any type of dietary modification. There are very few information provision interventions for pwMS that have been based on theoretical frameworks,^20^ despite this being recommended as best practice.^21^ Theoretical models are useful for understanding behaviour change and maintenance, and for developing interventions and strategies for behaviour change.^22^ One example is the self‐determination theory (SDT), which is a theory of human motivation, development, and health, focusing on the types of motivators as predictors of personal and well‐being outcomes.^23^ In the field of physical activity and MS, the concepts of SDT have been applied to better understand physical activity behaviours and adherence in pwMS.^24^, ^25^

Exploring the motivations and barriers for healthy dietary behaviours in pwMS would aid in developing evidence‐based dietary resources and interventions for pwMS. These should aim to help pwMS achieve national dietary guideline recommendations, thus reducing the risk of co‐morbidities and potentially improving quality of life.^26^ The aims of this research were to explore the experiences of adults with MS when navigating dietary advice, their attitudes when making dietary decisions, and their needs regarding dietary resources and education.

## 2 METHODS

This study was approved by the Human Research Ethics Committee at Curtin University (approval HRE2019‐0179). Given the paucity of qualitative literature in the field of diet and MS, we used a general inductive approach to guide this research, where themes were derived from interpretations of the raw data^27^ and reviewed for connections to theoretical frameworks.^28^ This allowed the analysis to be guided by the objectives and ensured participant responses were not influenced by predetermined hypotheses.^29^ Focus groups were conducted between July 2019 and March 2020 in Western Australia. The research information statement (outlining the study aims, expected duration and anonymity) was provided to participants before the focus groups commenced. Participants provided written informed consent. We adhered to the Consolidated Criteria for Reporting Qualitative Research (COREQ).^30^

### 2.1 Participants and recruitment

Participants were eligible for inclusion if they were English‐speaking adults (age ≥18 years) diagnosed with MS. There were no exclusion criteria. We used purposive sampling to recruit participants from a local MS organization (MS Western Australia [MSWA]) and networks of the project stakeholder advisory group (which included two MS consumer representatives). The study was advertised by MSWA via emails to the member database and social media postings. Potential participants were invited to take part in a single focus group. Participants were given an AUD$20 department store voucher as remuneration. No participants withdrew from the study after attending a focus group.

### 2.2 Data collection

We aimed to conduct 5‐6 focus groups with 5‐8 participants per group. The focus groups were facilitated by RDR (nutritionist, BSc[Hons]), with one of either AB (dietitian, DrPH) or LJB (nutritionist, PhD) as co‐facilitators. The topic guide (Table 1) was developed with input from both the relevant literature^18^, ^31^, ^32^ and the research team, and the first focus group was used as a pilot group to test the suitability of the questions with participants who had consented. As a result of piloting, the topic guide was unchanged and therefore the transcripts from the pilot group were included in analysis. Participants were asked to arrive 30 minutes before the start time to establish rapport with other participants and the researchers, since the researchers did not have existing relationships with the participants. During the focus groups, probing was used to clarify information or seek further details. Demographic information (sex, age, type of MS and duration of MS) and nutrition program preferences (delivery mode, topics of interest, and frequency, duration and number of sessions) were collected using two short questionnaires developed by the research team. To maintain a reflexive stance,^33^ the facilitators discussed and made notes after each session to reflect on their assumptions and biases, and how their role as researchers influenced the group discussions. Memos documenting key phrases, states of mind, emotional responses and/or questions to probe in subsequent groups were written after each group. The focus groups were audio‐recorded and transcribed verbatim. Transcripts were posted to participants for member checking,^33^ confirming that data represented the group discussions. Focus groups were conducted until thematic saturation was reached (ie no new codes emerged).^34^

**TABLE 1:** Focus group topic guide

Topics	Discussion guide
Introduction	Welcome, purpose of the research, ground rules, format, anonymity reminder. Are there any questions before we begin?
Icebreaker	Thinking about the last week, has your MS impacted on what you are eating or what you've chosen to eat?
Barriers and facilitators	Can you tell me about any ways that you may find your MS affects the way you eat? [Probe: shopping and preparing food, use of utensils or equipment, cooking methods, side‐effects from medications, fatigue] What sort of things do you do that make it easier for you to eat well?
Dietary information or advice	What (if any) dietary information have you asked a health professional about? Whose role is it to give out information about diet/foods for MS?
Dietary education program preferences	What would you have liked to have known about food or diet when you first found out you had MS? What topics would you like covered in an MS nutrition program? What types of things would need to happen for you to know you had made improvements, and what improvements are important to you? Have you been to any seminars related to MS? If so, what did you attend, and what did you like and not like about those events?
Wrap‐up	Is there anything else about diet and MS that you want to talk about that we have not discussed?

### 2.3 Analysis

Transcripts were managed with NVivo (version 12.6.0, QSR International Pty Ltd). The first author used a general inductive approach^27^ to thematically analyse all transcripts. Analysis commenced after the first focus group. The initial coding stage involved two authors (RDR and AB) reading the transcripts in detail and labelling text relating to each of the objectives. RDR then labelled behaviours, strategies, and states of mind using literal (direct observations) and interpretive (inferred from the data) coding techniques,^35^ which included text unrelated to the objectives. This resulted in 31 initial categories. In the second stage of coding, 15 categories resulted from grouping those with similar meanings. Final revision of the data involved further grouping of categories with similar meanings and collapsing redundant categories. RDR and AB discussed the categories and emerging themes several times during the analysis as a form of peer debriefing. This produced six main themes^33^ which were confirmed by the research team. A secondary analysis was conducted by RDR, where the lens of the self‐determination theory (using the constructs of autonomy, competence and relatedness) were applied to explain the themes.

## 3 RESULTS

### 3.1 Participants

Thirty‐four participants (33 pwMS; one spouse) attended one of six focus groups. The mean number of participants per group was six (range, four to eight). Focus group duration was between 50 and 68 minutes (mean, 60 minutes). The majority of the participants were female (82%), and the mean (SD) age was 50.2 (12.4) years. The median time since diagnosis was 6 years (range, 0.5‐37 years), and the most common type of MS was relapsing‐remitting (68%). See Table 2 for participant characteristics.

**TABLE 2:** Participant characteristics (n = 34)

Sex, n (%)	
Female	28 (82.4%)
Male	6 (17.6%)
Age (y)	
Mean (SD, range)	50.2 (12.4, 27‐79)
Time since diagnosis (y)^a^	
Median (IQR, range)	6.0 (13.5, 0.5‐37)
Type of MS^a^ n (%)	
Relapsing‐remitting	23 (67.6%)
Secondary progressive	1 (2.9%)
Primary progressive	4 (11.8%)
Unsure or other	5 (14.7%)
Country of birth,^b^ n (%)	
Australia	21 (61.8%)
Overseas	11 (32.4%)
Employment status, n (%)	
Employed	17 (50%)
Disability pension	5 (14.7%)
Retired	6 (17.6%)
Other^c^	6 (17.6%)

Abbreviations: IQR, interquartile range; MS, multiple sclerosis; SD, standard deviation.

^a^ Participants with MS, n = 33 (one spouse attended one focus group).

^b^ Missing data, n = 2.

^c^ Other included home duties, looking for work, not working and volunteering.

### 3.2 Themes

Six themes emerged: (a) confusion about where to seek dietary advice; (b) scepticism towards national dietary guidelines; (c) personalized approaches to dietary change; (d) barriers to dietary changes; (e) judging if dietary changes work; and (f) wanting dietary guidelines for MS. Participant number, focus group number, and time since diagnosis are detailed after each quote.

#### 3.2.1 Theme 1: Confusion about where to seek dietary advice

Participants discussed accessing dietary information from a wide range of sources: friends, family, health‐care professionals, websites, documentaries and books. Dietitians and MS organizations were rarely mentioned. The conflicting information about diets for MS meant there was confusion about what were appropriate foods and diets. It was difficult for some participants to judge reliability; causing angst when deciding which foods include/exclude, or which specific diet to follow.

There are so many different diet plans and people having their two cents' worth on the internet, and it's like a minefield trying to get information that's relevant and correct. (P15, FG2, 6 years)

Should I go on Keto? Should I go on low‐fibre? Should I do this? Can I eat a low‐GI bread? Can I eat gluten‐free bread? […] I just have no idea. (P31, FG6, 20 years)

Some participants were afraid that their dietary decisions may cause a relapse and were anxious when deciding what to eat.

It can create a lot of anxiety because you're so frightened of, on one hand, of having a relapse. Which way do I go when there's no, um, official guidance. (P20, FG4, 2 years)

Participants indicated an interest in what other pwMS were doing with diet, seeking confirmation from their peers about their dietary modifications.

Can I ask, do you do gluten‐free? So I've always had this question mark over this, is this something? Why do you guys do gluten‐free? (P11, FG2, 6 years)

It was discussed that neurologists and other MS health professionals did not promote any specific diets for MS. Participants thought that neurologists generally had inadequate knowledge and/or training to give dietary advice, and their focus was on treating the disease with medication. Some participants were alarmed that neurologists were not interested in dietary modification as *‘preventative medicine’* (P24, FG4, 10 years), which is how some restrictive MS diets are promoted. There were some comments that were conspiratorial in nature: neurologists and other MS professionals were keeping something from them and could be sued if they recommended diets other than the national dietary guidelines.

I said surely diet's gotta be‐ play a big role in this sort of thing, right? And they‐ it was almost like they [neurologists] were barred from saying yes. (P10, FG2, 6 years)

They've [neurologists] got the guidelines, and they can't sway from it, otherwise, they get sued and all sorts. (P18, FG3, 6 months)

Despite the perceived lack of training and/or interest in diet by neurologists, the participants agreed that they wanted to receive dietary advice from their neurologists.

#### 3.2.2 Theme 2: Scepticism towards national dietary guidelines

Participants were sceptical as to whether the *‘national guidelines’* (P33, FG6, 4 years) or the *‘healthy food pyramid’* (P6, FG2, 2.5 years) were suitable for pwMS. In light of the information about diet and MS that participants were accessing online and in books, national dietary guidelines were perceived as not good enough. There were misconceptions about what was recommended within those guidelines, for example participants thought it was necessary to consume all foods in the guidelines, including meat, dairy and grains. Vegetarian and vegan diets were not considered to be compliant with national dietary guidelines.

On that food pyramid is dairy. Well, should we be eating dairy? Or should be substituting the dairy section? (P28, FG6, 17 years)

Some participants were frustrated and angry in response to being given the ‘national guidelines’ or ‘food pyramid’ as dietary advice. In some cases, there was scepticism about the suitability of the ‘national guidelines’ for the general population, as well as for pwMS.

It's not a healthy diet, even though you're eating your five pieces of wholemeal bread a day, and you know, your two cups of pasta, or whatever. […] The powers that be realised they made mistakes 40‐50 years ago when they came up with the National Dietary Guidelines. (P15, FG3, 6 years)

Absolutely. That pyramid is an absolute load of crap. (P18, FG3, 6 months)

#### 3.2.3 Theme 3: Personalized approaches to dietary change

The general discussion in the groups demonstrated that most participants were highly motivated to learn about potentially beneficial dietary modifications. Some participants mentioned that they were very strict when adhering to their dietary changes, many adopted a moderation approach, and a few did not make any dietary changes. During the discussions, it became evident to the research team that part of the reason for attending was to discover what other pwMS were doing with diet, and that modifying their diets was a way for pwMS to feel in control of their disease. Some participants were convinced it would slow disease progression and help to avoid disability.

It's something you feel you've got‐ that you can control […]. You can't control your MS, you know, but, you can control your diet. (P8, FG2, 37 years)

Sometimes participants were very persistent about the dietary approach they were taking and were open to sharing what had and had not worked for them. A wide range of dietary modifications were described, from small or targeted dietary changes (eg eating more fruits and vegetables, eliminating sugar, reducing fat intake and/or eliminating food groups), to total dietary changes (eg adopting a specific diet such as the Wahls Protocol diet,^36^ the Overcoming MS Recovery Program diet,^37^ the ketogenic diet^7^ or the Swank diet^7^).

My diet's changed in ways of being more aware […] Instead of going to KFC, you'll go and have a Subway because it's got salad and vegetables, and all that sort of stuff. Or you know, if you have takeaway stuff I'll have a kebab because it's got meat, it's got vegetables. (P25, FG5, 1.5 years)

I've been dairy‐free, sugar‐free, gluten‐free, eating nine cups of vegetables every day, sourcing you know, good quality veggies and good quality meats. Before that, I was just a regular person eatin' anything I wanted. (P18, FG3, 6 months)

Participants had different opinions about how strictly they thought they should adhere to their chosen diet, and about their capacity to sustain the changes. There was conversation about continuing to eat all foods *‘in moderation’* (P25, FG5, 1.5 years), predominantly from those who were more recently diagnosed.

I am just going to try and live my life right now, and get into a least some kind of healthy pattern. I'm not gonna cut out dairy, I'm not gonna cut out those things out. I'm just gonna be more realistic about the amounts […] and what's possible for me. (P4, FG1, 2 years)

For participants without many MS symptoms, food or diet was considered a low priority. They stated that they assumed that their neurologist would have informed them if a specific diet or dietary modification was important. Maintaining current dietary habits was a way of upholding some normality and, for some, represented a degree of denial about the perceived need to change and/or about their diagnosis.

I don't read about it [diet] […] I've just kind of ignored it. I'm a bit blasé about. […] Has anybody's neurologist even given them any advice on diet? It's not something I've looked into or thought about to be honest. (P29, FG6, 20 years)

I don't see any difference in my MS, so I don't‐ I haven't done a lot of research [about diet]. Like maybe there's still some blinders up. (P26, FG5, 1.5 years)

At the other extreme, some participants were very serious about their chosen dietary modifications to slow their disease progression or keep their symptoms at bay. Those participants believed that following a specific diet was of the highest priority, which required a lot of time and mental effort. The choice between strictly adhering to a specific diet or not was likened to choosing between continuing to be able to walk (not ending up in a wheelchair) or eating McDonalds (fast‐food).

Your future, it's everything. Like, if you wanna be‐ if you wanna, you know, eat McDonalds, or do you wanna walk? That's kinda like the choices I made. (P11, FG2, 6 years)

It appeared that the participants committed to personalizing their diet plans in an attempt to recognize the individuality of the disease, and to cope with the conflicting dietary information. Some dietary modifications described by participants were an amalgamation of diets, creating a so‐called *‘flexitarian’* (P20, FG4, 2 years) diet. As they discussed their eating habits in the groups, it was apparent that even those claiming to follow one specific diet were incorporating aspects from other diets. There did not appear to be any practical reasoning in the decision‐making process; rather a lucky dip as to what might work. As they listened to what others in the group were doing with diet, some participants were confused about which specific diet they were adhering to: *‘So it is really a keto [ketogenic] or Mediterranean [diet]’*. (P34, FG6, 6 years).

It's a matter of taking a bit of that, and a bit of that, and a bit of that, and a bit of that, and like‐ and just piling it all into one [diet]. (P7, FG2, 22 years)

You really just got to find something that suits you and I'm the same. I'm well‐ Paleo‐ish. I know about Wahls Protocol as well, but gluten and dairy are the main things. (P15, FG2, 6 years)

#### 3.2.4 Theme 4: Barriers to making dietary changes

Even when diet was a high priority, it was not always easy to achieve or maintain the desired dietary modifications. Since the majority were working, time to prepare ingredients and cook meals was limited by long days at work.

I live on my own as well, so to try and do all those things, and work full time, and get home […]. I'm generally pretty tired by the time I get home at 6:30 anyway. But that kind of impacts my food choices. (P4, FG1, 2 years)

The participants' living situations dictated the capacity to strictly adhere to a specific diet. Many did not want to cook two meals at each eating occasion (ie one meal for themselves, and one for their partner and/or the rest of the family). Rarely, participants described putting in the effort to cook separate meals.

I've got a family, so we can't [afford], for me to have a different diet. (P31, FG6, 20 years)

It was common for MS symptoms to present as a barrier to sticking to planned dietary modifications. Fatigue, feeling unwell and having a relapse, typically caused participants to waver from their dietary plans.

My fatigue is so high that if I'm at home on my own […] I can't be bothered cooking. […] I don't have the energy to get up and cook. (P13, FG3, 15 years)

Some of the specific diets required special ingredients that were expensive and only available at specialty stores. There was discussion on how managing a specific diet was ‘hard work’ (P10, FG2, 6 years) and required mental and physical effort every day.

Suddenly you have this whole obscure list of ingredients [ha], like where do I find this stuff anyway? Um, and then it costs like $22 or something for it, rather than paying, I don't know, $2.99 for cereal you're paying $14 for something different. (P4, FG1, 2 years)

The participants described ways in which they overcame some of the barriers to making dietary changes. Strategies to overcome fatigue included prepping raw ingredients during the day, cooking large batches of food to freeze, and using kitchen appliances such as slow cookers, mandolin slicers and food processors.

I've got a Thermomix, which I love, and it chops. It just does make my coleslaw in like seven seconds and all I have to do is you know just chop it into chunks and chuck it in. (P13, FG3, 15 years)

Assistance from family or MS support workers with shopping, preparing ingredients, and/or cooking reduced the effort and fatigue for some pwMS.

I'm so lucky to have MS helpers come […]. A food prep person that comes in once a week, and she makes a huge chicken broth that lasts a week. (P10, FG2, 6 years)

#### 3.2.5 Theme 5: Judging if dietary changes work

The presence or absence of MS symptoms was discussed as ways to judge the impact of dietary modifications. Changes in energy levels, limb strength or dexterity, cognitive clarity (*‘brain fog’* [P2, FG1, 6 years]), skin condition (eg pimples, hives or itchy skin), bladder and bowel functioning, and the presence of migraines were perceived to be the direct result of dietary modifications. Seeing an improvement in their symptoms motivated participants to continue with the dietary changes.

I think [it's] how we feel, like literally. You know if the fasting's going to help you with your symptoms or feeling um‐ I really gauge on how I feel. (P10, FG2, 6 years)

I find when I was eating bread and all that crap, that's when I had really bad issues with my bladder and bowel. So that's why I stopped. (P33, FG6, 4 years)

There was uncertainty about whether fluctuations in energy or mood were a result of dietary modifications, or if they were simply due to MS. Despite this doubt, participants were hesitant to revert back to old dietary habits.

I think I did actually feel better, but again I don't know if that's because I was just having a period of time that I felt better, or whether the diet changes made me feel better. (P4, FG1, 2 years)

[My] neurologist said 100% [that] the diet hasn't had any benefits to my MS at all, uhm, but like I've said to my husband, he can't prove to me that I wouldn't be worse if I wasn't eating healthier […] He can't tell me I wouldn't be ten times worse if I wasn't implementing the diet. (P21, FG4, 3 years)

There was some discussion that objective measures of MS progression, such as lesion activity evident from magnetic resonance imaging scans, gave definitive answers about the effectiveness of dietary modifications. This eliminated the need to make judgement calls based on feelings or symptoms. In those situations, it was not clear how the effects of dietary modification were differentiated from either the natural disease progression or benefits from disease‐modifying therapies.

My diet significantly changed […]. As a result, I haven't had an attack, no more new lesions. (P18, FG3, 6 months)

One participant described experiencing a relapse after making a dietary modification. This was considered to be evidence that diet does influence disease progression.

The time that I slipped was when um, I got introduced to coconut oil as a good fat, and I ended up relapsing. (P11, FG2, 6 years)

#### 3.2.6 Theme 6: Wanting dietary guidelines for MS

Despite being sceptical of the national dietary guidelines and personalizing specific diets, participants overwhelmingly wanted to be told what to eat for their MS. While they accepted that guaranteed benefits were unlikely, participants wanted to know what dietary modifications *may* be beneficial. The desire for clear MS‐specific dietary advice contradicted the discussions about personalizing diet plans. While participants agreed that they wanted to be told what to eat, the individuality of MS meant that *‘a one‐size fits‐all’* (P29, FG6, 20 years) answer for diet was unlikely.

You just want someone to say‐’ (P4, FG1, 2 years) ‘‐eat this, or do this, and this will make it, make your life better. […] (P5, FG1, 6 years)

I think you go, okay, “dairy‐free, gives you these benefits because it affects, I don't know, your gut, or your acidity, or your inflammation, or your fatigue.” […] “If you go dairy‐free then this is the benefits you should feel because we've researched it.” (P28, FG6, 17 years)

I don't think you could tell somebody that this is the diet for MS, because we're all so different. (P34, FG6, 6 years)

There was discussion about the desire for MS‐specific dietary guidelines: a *‘pyramid chart to show you what's accurate for MS*’ (P10, FG2, 6 years). The participants wanted well‐researched *‘baseline’* (P30, FG6, 16 years) guidelines, which could be adapted to suit their own personal experiences with diet. The national dietary guidelines were not seen to fit this need.

We all understand that whole triangle [food pyramid]. But on that triangle are things like dairy, wheat, pasta, rice. […] Should our food pyramid be substituted with “okay, instead of eating this, eat this.” This is our food pyramid because science, food science, tells us that for our gut we don't eat the dairy, we don't eat the wheat. (P28, FG6, 17 years)

Participants wanted simple instructions about suitable dietary modifications for MS, including a *‘list of foods to avoid [and a] list of foods to eat*’ (P15, FG2, 6 years). Access to evidence‐based MS‐specific dietary guidelines would provide relief from having to sift through the *‘minefield’* (P15, FG2, 6 years) of information on the internet. MS‐specific dietary guidelines would give participants the confidence and motivation to adhere to dietary changes, since they would be sure that they were meeting their nutritional requirements. Participants agreed that an MS dietary education program would be ideal to learn about MS‐specific dietary guidelines, and that it should be facilitated by a credible health professional (nutritionist or dietitian).

## 4 DISCUSSION

This qualitative study provides insight into the experiences of pwMS when navigating dietary advice, their attitudes when making dietary decisions, and their needs regarding dietary resources and education. PwMS were confused about where to seek dietary advice. The majority of participants thought that neurologists were not allowed to counsel on specific diets outside of the national dietary guidelines and were sceptical about the suitability of the guidelines for pwMS. Most pwMS were highly motivated to make dietary modifications, and they wanted MS‐specific dietary guidelines.

Why pwMS make dietary modifications—and their varying levels of motivation—can be explained by the central tenants of SDT.^23^ There are three fluid types of motivation on a continuous scale. At one end is amotivation, where there is a lack of motivation to change. For some pwMS, not getting MS‐specific dietary advice from their neurologist led to greater amotivation. Further along the scale is controlled or external motivation, where behaviour change is shaped by extrinsic factors, such as obligation or coercion. Health professionals could play a role in motivating patients by providing this type of motivation. For pwMS, the perceived physical benefits for disease management (an external motivation) from physical activity has been reported as a positive predictor of physical activity participation.^24^ At the other end, the highest form of motivation is autonomous motivation, which occurs when the values of an activity have been integrated into one's own values.^23^ This type of motivation has been associated with improved physical and psychological outcomes.^38^ The desire to achieve intrinsic goals (eg improving health through dietary behaviours, and the accompanying sense of accomplishment) meets the needs for autonomy and competence in pwMS to drive behaviour change.^23^ A nutrition education program could provide information and skills that also meet those needs.

Seeking information and maintaining dietary behaviour change were driven by external motivation. In the group discussions, the participants were eager to know what their peers were doing with diet, even if they had not made dietary changes themselves and/or were encouraging each other to consider dietary modifications that seemingly worked for themselves. For some, the fear of worsening symptoms (which causes worry, and disrupts valued activities and everyday routines of pwMS^39^) or relapse due to dietary behaviours was a form of external motivation to continue with their dietary modifications. Similarly, external motivation has been associated with adherence to dietary recommendations in people newly diagnosed with type 2 diabetes.^40^ When discussing where to seek dietary advice, the participants in our study were confused and found it difficult to determine what information was credible. They wanted dietary advice from their neurologists (external motivation). These findings are in line with our previous study of people newly diagnosed with MS, who found it difficult to judge the credibility of dietary information and wanted input from their neurologists.^18^

Most of the participants in our study were highly motivated to make dietary modifications, and their behaviours were autonomously motivated. The participants' goals to manage their symptoms and improve their health were intrinsic, since diet was something they could change to self‐manage their disease. This autonomous motivation was driven by the apparent ability to make and adhere to dietary modifications, and the perceived effectiveness of those changes (ie improvement in MS symptoms). When autonomous or internalized motivations drive behaviour change, the outcomes are more sustained.^23^ Making dietary modifications is a way that pwMS can feel in control of their disease,^18^, ^41^ and provides a sense of hope since they are able to take action.^42^ Newly diagnosed pwMS prioritize their health (motivations are autonomously driven)^41^; hence, this is an ideal time to help pwMS build competence and improve their diets to meet the recommendations of the national dietary guidelines.

Focus group discussions revealed that participants wanted to be told what to eat for their MS. MS‐specific dietary advice from neurologists (the first MS health professional encountered after diagnosis), appropriate referral to dietitians, and access to an evidence‐based nutrition education program could give pwMS the confidence and motivation to make and adhere to dietary modifications. Credible and evidence‐based dietary advice from a neurologist could start to build intrinsic motivation for pwMS and empower them in their decision making^43^ and provide an additional source of external motivation. An MS nutrition education program that is based on national dietary guidelines and highlights potential benefits for pwMS could contribute to building self‐esteem and autonomy in making dietary decisions, and provide pwMS with the tailored education they seek (ie nutrition education for MS); as opposed to the generic advice that they report receiving from their health‐care providers.^18^ Over time, this could transition motivation from external to autonomous, as the drivers for dietary behaviour change become more intrinsic. This could lead to pwMS making healthier choices, since autonomous forms of motivation are central to the adoption and maintenance of healthy diets.^44^

A strength of this study was that we included participants who had MS for varying lengths of time, and the proportion of females compared with males was similar to the sex distribution of the disease. Our study has some limitations. The participants who chose to participate may have been more motivated to make dietary modifications than the typical population of pwMS, resulting in selection bias. People who had low English proficiency, such as culturally and linguistically diverse groups, may not have participated. The views expressed were representative of the participants, but may not be generalizable to the wider MS community.^33^ There is the potential for social desirability bias, where participants may have conformed to the general group consensus, instead of expressing their authentic views.

## 5 CONCLUSION

People with MS want to take action in the self‐management of their disease, and they are reconsidering their lifestyle choices after diagnosis. Evidence‐based MS‐specific dietary resources need to be available from dietitians and neurologists. Such resources should highlight the potential benefits from adhering to national dietary guidelines, for example avoiding nutrient deficiencies that may exacerbate MS symptoms. Given the suitability of the SDT for explaining how pwMS make dietary decisions, and the different degrees of motivation for dietary change, future dietary change interventions could use the SDT as a framework for design. Our finding that people tend to personalize specific diets promoted for pwMS is informative for future quantitative research: surveys need to provide participants with the opportunity to detail the dietary changes they are adhering to, including any variations to specific diets promoted for pwMS.

## CONFLICT OF INTEREST

The authors declare no conflicts of interest.

## PATIENT OR PUBLIC CONTRIBUTION

Members of a stakeholder advisory group, which included a neurologist, MS counsellor, MS nurse, psychologist, dietitians and MS consumers, provided input during the development of the question guide and methods for participant recruitment. One MS consumer provided feedback after the pilot focus group session.

## Data Availability

The data that support the findings of this study are available from the corresponding author upon reasonable request.
